# Motivation is not enough: A qualitative study of lung cancer screening uptake in Australia to inform future implementation

**DOI:** 10.1371/journal.pone.0275361

**Published:** 2022-09-30

**Authors:** Kate L. A. Dunlop, Henry M. Marshall, Emily Stone, Ashleigh R. Sharman, Rachael H. Dodd, Joel J. Rhee, Sue McCullough, Nicole M. Rankin

**Affiliations:** 1 Daffodil Centre, The University of Sydney, A Joint Venture with Cancer Council NSW, Sydney, NSW, Australia; 2 Department of Thoracic Medicine, The Prince Charles Hospital, Brisbane, QLD, Australia; 3 The University of Queensland Thoracic Research Centre, Brisbane, QLD, Australia; 4 Department of Thoracic Medicine and Lung Transplantation, St Vincent’s Hospital, Darlinghurst, NSW, Australia; 5 School of Public Health, Faculty of Medicine and Health, The University of Sydney, Sydney, NSW, Australia; 6 School of Clinical Medicine, Faculty of Medicine and Health, UNSW Sydney, Kensington, NSW, Australia; 7 School of Population Health, Faculty of Medicine and Health, UNSW Sydney, Kensington, NSW, Australia; 8 Graduate School of Medicine, Faculty of Science, Medicine and Health, University of Wollongong, Wollongong, NSW, Australia; 9 Lung Foundation Australia, Milton, QLD, Australia; 10 Centre for Health Policy, Melbourne School of Population and Global Health, The University of Melbourne, Melbourne, Vic, Australia; University of South Australia, AUSTRALIA

## Abstract

**Introduction:**

Participation in lung cancer screening (LCS) trials and real-world programs is low, with many people at high-risk for lung cancer opting out of baseline screening after registering interest. We aimed to identify the potential drivers of participation in LCS in the Australian setting, to inform future implementation.

**Methods:**

Semi-structured telephone interviews were conducted with individuals at high-risk of lung cancer who were eligible for screening and who had either participated (‘screeners’) or declined to participate (‘decliners’) in the International Lung Screening Trial from two Australian sites. Interview guide development was informed by the Precaution Adoption Process Model. Interviews were audio-recorded, transcribed and analysed using the COM-B model of behaviour to explore capability, opportunity and motivation related to screening behaviour.

**Results:**

Thirty-nine participants were interviewed (25 screeners; 14 decliners). Motivation to participate in screening was high in both groups driven by the lived experience of lung cancer and a belief that screening is valuable, however decliners unlike their screening counterparts reported low self-efficacy. Decliners in our study reported challenges in capability including ability to attend and in knowledge and understanding. Decliners also reported challenges related to physical and social opportunity, in particular location as a barrier and lack of family support to attend screening.

**Conclusion:**

Our findings suggest that motivation alone may not be sufficient to change behaviour related to screening participation, unless capability and opportunity are also considered. Focusing strategies on barriers related to capability and opportunity such as online/telephone support, mobile screening programs and financial assistance for screeners may better enhance screening participation. Providing funding for clinicians to support individuals in decision-making and belief in self-efficacy may foster motivation. Targeting interventions that connect eligible individuals with the LCS program will be crucial for successful implementation.

## Introduction

Lung cancer is the leading cause of cancer death worldwide [1] including in Australia [2]. Lung cancer survival in Australia remains poor compared to other cancers and has improved little over time, increasing from 9.5% to 19% between 1987–1991 and 2012–2016, despite a reduction in smoking rates (11% of the population aged 14 years and over reported smoking daily in 2019) [3]. Early detection is the key to improving patient outcomes [4, 5]. Large international trials [6, 7], have demonstrated significant reductions in lung cancer mortality with low dose computed tomography (LDCT) chest screening in individuals at high-risk of developing lung cancer. Yet, low uptake of lung cancer screening (LCS) in those at high-risk has been reported in clinical effectiveness trials [8] and screening programs [9]. Many participants opt out of baseline screening after registering interest [10, 11]. Tobacco smoking, a highly stigmatised behaviour, is the single biggest risk factor for lung cancer [4] and is highly correlated with socio-economic status, ethnicity, education level and geographical remoteness[12]. Consequently, potential LCS participants are often identified as ‘hard-to-reach’, i.e., having limited capacity for involvement or are difficult to involve in participation of health programs [13]. These factors present challenges for successful implementation of targeted, organised LCS programs.

Previous research suggests that individuals with current or former smoking histories are willing to undergo LCS [14, 15] especially if the screening centre is nearby [15, 16], is recommended by a clinician [17, 18] and perceived to have potential improvements in health outcomes [15, 19]. However multiple barriers impact on the decision to participate in LCS [17, 20]. Blame and stigma around lung cancer as a self-inflicted smokers’ disease [15, 21], worry about lung cancer risk [21], cost, distrusting the medical system [22], false positive results and fear of excessive radiation exposure [23] have all been reported. Poor uptake of screening in lower socio-economic communities has also been attributed in part to fearful and fatalistic beliefs related to a high level of exposure to lung cancer and respiratory conditions [24]. In addition, the value of screening in a high-risk population has been questioned by potential participants [21] and professional organisations [25]. In one UK study, participants from lower socio-economic communities suggested screening may not prolong the lives of the older generation of ‘heavy smokers’ [21]. Although opting out of screening in a hypothetical scenario did not appear to be influenced by worry about false-positive results or lung cancer risk in an Australian study [16], these factors were influential in a real-life study [10].

Factors that are associated with enabling or facilitating participation in lung cancer screening are less clear cut. Participation in recent LCS trials is linked with belief in screening efficacy [26, 27], trust in medicine and high self-efficacy [18]. However, Carter-Harris et al [18] noted screening behaviour and self-efficacy appeared to be fully mediated by a range of factors including fatalism and knowledge of lung cancer risk. In addition, while clinician recommendation had significant impact on screening decisions in several studies [18, 27], participant’s attitudes towards healthcare provider recommendations vary across settings and do not always facilitate screening uptake [26].

Some LCS studies identify strategies that may influence participant screening behaviours [17, 24, 28, 29]. In practice, effective intervention design requires identification of the context and specific target in the population [20]. Understanding what drives behaviour from the perspective of the individual who has participated or declined screening may help inform these target points. Qualitative research is ideally suited to exploring individual perspectives [30], particularly when well targeted. Interview questions informed by the Precaution Adoption Process Model (PAPM) ensure participants are targeted at the appropriate stage ‘decision to act’ [31]. PAPM, a stage theory, recognises that individuals are at different points in the process of adopting preventive behaviours and that interventions that influence action will vary across stages [31]. We used two theories, the PAPM for interview question development and the COM-B theory of behaviour change given our focus on and commitment to Implementation Science principles, which emphasise the importance of all research being underpinned by relevant theories, models or frameworks. Analysis using a behavioural model enables focus on the understanding of behaviours and identification of potentially modifiable factors that could encourage behaviour change [32]. The COM-B (Capability, Opportunity, Motivation-Behaviour) model suggests that behaviour involves the interaction of these components. While motivation is central, capability and opportunity influence the relationship between motivation and behaviour. The greater the capability and opportunity, the more likely the behaviour is to occur [32, 33].

Currently there is no national lung cancer population screening program in Australia. Recently, the Australian Government invested in scoping the feasibility of a potential LCS program [2] that aims to detect disease at an early stage and avoid any out-of-pocket payments for participants. Understanding the factors that drive screening participation will be crucial for successful implementation [4, 12]. This qualitative study explores the perspectives of high-risk individuals within an Australian cohort who were eligible for screening and had either participated or declined to participate in the International Lung Screening Trial [34, 35]. Specifically, we aimed to identify the potential drivers of screening participation in the Australian setting, to inform future implementation of LCS.

## Method

### Participants

Qualitative interviews were conducted with LCS-eligible individuals at two Australian sites of the International Lung Screening Trial (ILST). Individuals were invited to participate in semi-structured telephone interviews approximately two years after being assessed for the ILST screening study. Interviewees were categorized as ‘screeners’ (had completed a baseline LDCT scan within the last two years) and ‘decliners’ (LCS-eligible individuals who declined to be screened).

Recruitment to ILST followed a three-step informed consent procedure[34, 35]: Step 1) potential participants registered interest for the ILST in response to advertisements and press releases by leaving a voicemail message at the research office; Step 2) research staff telephoned potential participants to explain the trial and share information about the benefits and harms of lung cancer screening; potential participants had the opportunity to ask questions of a lung cancer nurse over the telephone; eligibility for screening was assessed during this phone call; Step 3) eligible participants (see below) who were interested in LCS were sent a participant information sheet and were required to provide written informed consent prior to screening. ILST did not include a shared decision-making component over and above written informed consent. Participant eligibility was determined if they met the USPSTF2013 criteria (aged between 55 and 80 years, current or former history of smoking of at least 30 pack-years) and/or had a 6-year lung cancer risk of > = 1.51% using the PLCO_m2012_ risk assessment tool [35, 36].

Recruitment to the qualitative interviews was purposive with the aim of including participants with a range of risk characteristics, including highest PLCO_m2012_ risk scores for decliners, ethnicity and geographic remoteness. Initial invitations to participate were sent via text messages and participants who responded were then emailed or mailed an invitation letter, information sheet, and consent form to be returned by email or reply-paid envelope prior to the interview. Written participant consent was initially required however an option of verbal consent was provided during the COVID-19 pandemic due to restrictions on participants to print, scan and/or post a reply. A verbal consent script was prepared, read aloud verbatim and participant consent was recorded. This research was approved by The Prince Charles Hospital Human Research Ethics Committee, Brisbane (Project ID 66834) and the St Vincent’s Hospital Human Research Ethics Committee, Sydney (2020/ETH02309).

### Study design

Separate interview guides were developed for screeners and decliners (included in S1 File), informed by the Precaution Adoption Process Model (PAPM) [31]. During the interview, participants were asked a range of questions about how they felt about LCS, their reasons for attending or declining and factors that encouraged and discouraged screening attendance. All interviews were conducted by one researcher (K.L.A.D) and lasted between 13 and 50 minutes (mean = 27 minutes); interviews were slightly longer overall for decliners by 2–3 minutes. At both sites, interviews were first conducted with decliners until saturation was reached; screeners were then invited to participate. Participants were reimbursed for their time with a $50 AUD gift voucher.

### Data analysis

Interviews were audio-recorded, transcribed verbatim and analysed using the COM-B model [30]. Files were managed in NVivo 12 software (QSR International, Australia). Initially, four investigators (K.L.A.D, N.M.R, A.R.S, R.H.D) familiarised themselves with data by reading four transcripts, both decliners and screeners, and participated in discussion to identify key features. A further four transcripts were then coded independently by two investigators (K.L.A.D & N.M.R) (approximately 20% of the data) using a deductive approach mapping onto the COM-B model core constructs: capability (physical and psychological), motivation (automatic and reflective) and opportunity (physical and social). Remaining transcripts were coded by one investigator (K.L.A.D). All investigators contributed to the final analysis and met on three instances to review and adjudicate discrepancies around themes. This study has been conducted, designed and reported in accordance to the COREQ (Consolidated criteria for Reporting Qualitative research) checklist [30].

## Results

Thirty-nine participants were interviewed with screeners (25 of 115, 22%) and decliners (14 of 157, 9%) taking part. All participants were aged between 55–80 years, had a current or former history of smoking and 21/39 lived outside of a major city. Overall, the socio-economic index (SEIFA score which ranks areas in Australia according to relative socio-economic advantage and disadvantage) was slightly below the national average (mean: 993.63) and higher for screeners. However, the socio-economic index was higher for decliners from Sydney with two participants from the most advantaged quintile. Table 1 summarises the participant demographics.

**Table 1:** Demographic characteristics of participants.

		Participants who declined to screen: decliners (n = 14)	Participants who were screened: screeners (n = 25)	TOTAL N = 39
**Recruitment**	Site 1	9/98 (9.2%)	13/56 (23.2%)	22 (56.4%)
	Site 2	5/59 (8.5%	12/59 (20.3%)	17 (43.6%)
	Total	14/157 (8.9%)	25/115 (21.7%)	
**Gender**	Female	5	7	12 (30.8%)
	Male	9	18	27 (69.2%)
**Smoking status**	Currently smoke^a^	5/14 (35.7%)	8/25 (32%)	13 (33.3%)
	Stopped smoking^b^	9/14 (64.3%)	17/25 (68%)	26 (66.6%)
**Remoteness** ^**c**^	Major cities	9	9	18 (46.2%)
	Inner/outer regional	5	8	13 (33.3%)
	Remote/very remote		8	8 (20.5%
**Ancestry**	Non-European		2	2 (5.1%)
**Socio-economic index [SEIFA score]** ^**d**^ ^,^ ^**e**^	Mean Site 1	927.55	956.30	
	Site 2	1051.6	1039.42	
	Min, Max Site 1	779,1088	776,1096	
	Site 2	902,1133	736,1169	
	Quintile 1 (most disadvantaged)	5		
	Quintile 2	3	4	
	Quintile 3	0	7	
	Quintile 4	3	1	
	Quintile 5(most advantaged)	3	6	

^a^ **Currently smoke:** an individual currently smoking with a smoking history **≥** 30 pack-years where pack-year is defined as number of cigarettes per day/20 x number of years of smoking

^**b**^**Stopped smoking:** an individual who has previously smoked and has stopped for >1 year and quit ≤ 15 years ago

^c^Australian Statistical Geography Standard Remoteness Structure, 2016

^d^ Area-based index of relative advantage and disadvantage

^e^ National average SEIFA score = 1000 (Standard deviation [SD] 100)

Themes that were identified across all interview transcripts are reported under the constructs of the COM-B model. The coding framework is shown in Table 2 and included in S1 Table with definitions. The only marked disparity in responses about motivation between screeners and decliners was self-efficacy. However, there were a number of differences in relation to physical capability, psychological capability, physical opportunity and social opportunity.

**Table 2:** Coding framework: Drivers of lung cancer screening participation in Australia using the COM-B (capability, opportunity, motivation-behaviour) model.

COM-B construct	Framework	Themes
**Capability**	Physical	Ability to attend^a^
	Psychological	Knowledge and understanding^a^
**Motivation**	Automatic	Impact of lived experience
		Fatalism
	Reflective	Awareness of own risk
		Screening as beneficial
		Self-efficacy^a^
**Opportunity**	Physical	Location as a barrier^a^
	Social	Support from family^a^
		Stigma is ever present
		Access to a General Practitioner

^a^decliners and screeners differ

### Capability

#### Ability to attend

Participants were required to be healthy and asymptomatic to be eligible, thus current health concerns were rarely expressed as barriers to attend screening. However, carer’s responsibilities and short-term illness interfered with some decliners’ capability, which were in part related to physical ability to attend. For example, one participant reported she had a short-term illness: *“actually I got the flu before I was supposed to come down to participate in (screening)*.. *I was quite sorry that I couldn’t participate*, *I mean*, *I would have been keen to be involved” (PD14*, *decliner*, *female)*. Another participant explained his wife had been hospitalised: *“She (my wife) was in the hospital*, *and I was there every day*, *and it was just very inconvenient for me to go (for screening)… because I had to be with her*. *It was very hard and stressful” (PD5*, *decliner*, *male)*.

#### Knowledge and understanding

Some differences were observed between screeners and decliners relating to the theme: *knowledge and understanding*. Both groups had been provided with information about lung cancer screening and their eligibility for screening during the initial ILST recruitment process. Screeners and decliners indicated throughout the interviews that they had sufficient knowledge to process decisions around attending screening and many explicitly talked about how they weighed up the pros and cons. However, some decliners indicated they had a poorer understanding of screening. One participant attributed potential harms of screening radiation to their reason for declining: *“they said it was a bit of a higher level of radiation to have*, *and then I thought that might cause lung cancer or something that’s kind of why I pulled out of fear” (P10*, *decliner*, *female)*. Some participants indicated they misunderstood that screening involved a low-dose CT scan rather than an x-ray and the potential advantages, as one reported: “*You don’t mind doing that on a holiday (travelling all the way to Brisbane) but I’ve never done it and to start doing it for medical treatment when I’ve got X-ray machines up here’ (P8*, *decliner*, *male)*. Similarly, another explained: *I don’t know what the fundamentals of getting your lungs screened*. *I wouldn’t have a clue*. *I just thought getting x-rays*, *that’s all (P9*, *decliner*, *male)*.

One participant, aware of being high-risk but who declined to attend, explained that feeling healthy and the challenges of long-distance travel outweighed his need for screening: *“it just got too difficult for me*. *it’s just too far*, *like it’s 700 kms*. *Not that that worries me normally*, *we’ve done a few big caravan trips but I thought*, *well*, *I think I’m pretty healthy” (P1*, *decliner*, *male)*. Here, the participant identifies that the very nature of population screening targeting the apparently healthy population often leads to a misunderstanding of its purpose.

### Motivation

Overwhelmingly participants expressed a relatively high level of motivation to participate in LCS, despite decliners opting out after their initial interest. Both groups reported similar perceptions and experiences about wanting to screen, except in relation to self-efficacy where decliners expressed a lower belief in their capacity to attend screening and execute healthy behaviours. The predominant motivating themes are described below.

#### Impact of lived experience

Discussion around the experience of caring for family or friends with lung cancer focused on its debilitating nature, challenging end-of-life experiences and the consequent importance of early detection. The lived experience was described by both groups with about one third (14/39) reporting a family history. One participant said succinctly: *“I think if you have lung cancer in the family*, *I don’t think anyone can tell you*, *I think you just do it” (P20*, *screener*, *female)*. Another participant, whose son had died of lung cancer, acknowledged the importance of early detection despite not participating in screening: *“That’s … one of the reasons I was going*… *My son*, *when he was discovered*, *it was stage IV (lung cancer)…I don’t know if they can save you if you find it early*, *and that’s obviously what I was doing it for*, *to see if I had any early signs of lung cancer*. *As I said*, *if you get to stage IV*, *that’s (the) end” (P4*, *decliner*, *male)*.

Similarly, another participant explained her personal motivation was related to her perceived lack of awareness of early detection and the impact on caring for her uncle: *“I have an Uncle*.. *he was 73 when he died of lung cancer*. *And he lived with me for about 20 years*, *he was like a father to me…*.*And*, *you know*, *I wished I had of known to recognise a sign*. *I didn’t*. *I just thought it was old age” (P36*, *screener*, *female)*.

### Fatalism

For other participants, although many expressed worry about their risk of lung cancer at times, this concern appeared to be minimal. Fear of receiving positive results was not seen as a barrier to screening and both groups were often fatalistic about developing lung cancer. *“Sometimes I do (worry about getting lung cancer)*, *but I haven’t stopped smoking permanently so*. *It’s just one of those things*, *I guess I’m going to get lung cancer or get run over by a bus*. *Who knows*?*” (P39*, *screener*, *female)*

Similarly, one participant who declined to attend as she had been unwell expressed a level of acceptability about her future health: *“I might think about it (risk of lung cancer) once every couple of months or something pops up on telly*. *But it’s not something that’s dominant*. *I mean*, *I do sort of probably focus on the fact that my time in this world is finite*. *And I don’t look at it in a morbid way*. *I just look at the practicalities of life” (P14*, *decliner*, *female)*.

### Awareness of own risk

Both screeners and decliners who reported a perceived increased risk of developing lung cancer acknowledged it was most likely related to their history of smoking, and for more, because of family history, providing a strong reason to attend screening. As another participant said: *“my mother died of lung cancer*, *I think it was back in 2009*. *And I’ve been smoking on and off since I was 16*. *So basically*, *I’m a long-time smoker and I wanted to do it to detect because I think I’m at high risk” (P39*, *screener*, *male)*. Similarly, a participant’s daughter identified her mother’s risk: *“Well I saw it on the news and I discussed it with my daughter and she said she*..*thought that it might be good for me because I’m a heavy smoker and my dad passed away with lung cancer” (P10*, *decliner*, *female)*.

#### Screening as beneficial

Motivation to attend screening was attributed in a large part to how highly screening was valued. All participants saw screening as beneficial. Some reported it as a means to stop lung cancer before it starts and others for early diagnosis leading to improving treatment outcomes and prolonging life: *“And of course if you detect it beforehand*, *there’s more chance of being able to cure it” (P39*, *screener*, *female)*. Another participant explained: *“I’m keen to have some sort of examination of the lungs in case*, *you know*, *there is something that can be to contribute towards a bit longer life…I do want to hang around for a while*…*(but I’m fifteen hundred kilometres away*.. *and I thought how the hell am I going to get to Brisbane)*.*” (P8*, *decliner*, *male)*

Screening was also seen as providing an opportunity to find answers to often unanswered questions about family history of lung cancer. One participant explained: *“My main motivation (to attend screening) is that my mother died of lung cancer*. *And she was never asked any questions*. *I did expect some questions to be asked*, *some investigation or research as to why maybe she did have lung cancer but that never happened*. *So my mother didn’t put a cigarette in her mouth between her lips in her entire life*. *(P28*, *screener*, *female)*

#### Self-efficacy

Typically, screeners expressed a greater sense of personal control and belief in their capacity to execute healthy behaviours and attend screening than decliners. One participant explained how she made the decision: *“I just told my husband*, *didn’t tell anyone (else)*. *I just did a lot of reading up about it*. *So*, *I sort of prepared myself for risks*, *benefits… it wasn’t a big decision to make*.*” (P13*, *screener*, *female)*

Participants’ sense of their own personal control often influenced the nature of the discussion. Many screeners reported confidence in their ability to reduce the risk of lung cancer: *“I live out on a rural property now*, *so I’m not even breathing in other people’s traffic (fumes)*. *I just have lots of fresh air and I don’t smoke*.*” (P18*, *screener*, *female)*

In contrast, decliners often expressed self-doubt and lacked confidence in attending screening. One participant reported how overwhelming the notion of travelling to an unknown location had been (although he lived less than 30 kms from the screening location) and anxiety associated with the screening process. He explained his reason for declining to attend: *“Which was going to take God knows how long to get there*. *And I have no idea where the Hospital is*, *and I didn’t know where I was going to park*, *and I was worried about my pacemaker*.*” (P4*, *decliner*, *male)* This lower level of self-efficacy was also apparent in some decliners who noted the challenges of giving up smoking. One participant said: *“I got to wake up myself and give up the smoke*, *so I try and try*, *and I say I’m not going to buy anymore again*, *but unfortunately*, *I do*.*” (P5*, *decliner*, *male)*

Participants in both groups, particularly screeners, felt it was important to be self-reliant and the final decision to screen was their own: *“It’s a very private thing*.. *It’s not up to anybody (else) to make up their mind that you can or cannot go” (P25*, *screener*, *male)*

### Opportunity

Participants described several factors related to physical and social opportunity that influenced their decision to attend screening. In contrast to screeners, decliners tended to report these factors as challenges summarised in the following themes.

#### Location as a barrier

For many decliners the distance required to travel to screening, unfamiliarity with the location and/or the cost of travel and accommodation near the screening site were the main reasons they didn’t attend screening. One participant from regional Australia explained: *“I booked in for it but it just sort of became too expensive*. *For me to go to the city*, *I’ve got to get a train down*, *train back*, *accommodation overnight*, *cab fares or bus fares*,.. *I don’t know my way around [the city of] Brisbane really well” (P12*, *decliner*, *female)*. Similarly, a participant who lived in the city where screening was located, declined screening and reported: “*So the reasons I didn’t go was because I didn’t know where the hospital was and I didn’t know where I was going to park” (P4*, *decliner*, *male)*

In contrast, a participant from the same city who attended screening reported that travel was not a concern: *“I got a bus into the city and a bus down to the hospital*. *Actually I got two buses*, *… a little less than an hour commute from door to door” (P22*, *screener*, *male)*. Many screeners who lived in regional or rural areas did not consider long distance travel as a hinderance and some reported added value in making the trip: *“When we live in these remote areas we don’t think distances are long and say*, *oh*, *we’re going*, *we flew down to the city for that initially*, *but we go on what we call our little road trip*. *And we’ve got family and friends dotted along the coast and we make a road trip out of it and catch up with everybody” (P33*, *screener*, *female)*

#### Support from family

Some participants reported that making the decision to participate in screening was a challenge in the absence of family support. One decliner explained how family relationships prevented the chance to discuss screening or for encouragement to attend: “*I think I don’t have much of a family anymore*. *And I’m not as close as I would like to be with them*. *And my wife*, *she’s okay with English*, *but not the greatest speaker… there’s a limitation to just how much I can converse with her in regards to screening”*…*(P8*, *decliner*, *male)*

Another decliner who lived by herself described the impact of the lack of support as a result of her family situation: *“I’d go and see my grandson down that way but*..*my son and his partner are separated*. *I haven’t seen my grandson now for nearly two years*, *which is heartbreaking*. *Even when I used to*, *it was a 17 hour trip for me because she (my daughter in-law) never invited me to ever stay there overnight*. *So it’s not something that I can get down there” (P12*, *decliner*, *female)*

While participants were quick to point out that the decision to attend lay with them, many screeners gave examples of encouragement and support from partners when feeling reluctant. One participant said: *“My wife particularly would have pushed me out the door*, *you know*, *go*, *now” (P27*, *screener*, *male)*. Similarly, another explained: *“I said to my wife*, *I’m a bit concerned about the dosage of radiation*, *and I’m thinking of maybe keeping out of this*. *And she said*, *well*, *you signed up for it*. *You know*, *you should keep going*. *And that sort of made me think again*, *too*. *And she was right” (P16*, *screener*, *male)*.

#### Stigma is ever present

The theme of stigma was most closely aligned with social opportunity. Most participants felt that lung cancer and smoking were seen as intertwined and that consequently there was considerable stigma attached to lung cancer. Both groups reported that generally the community associates the cause of lung cancer with smoking. One participant explained:

*“I think lung cancer is stuck fast to smoking*. *So whenever lung cancer is mentioned*, *people think of an ashtray and people who are smoking” (P17*, *screener*, *male)*. Another noted how blame was often placed on the individual who develops lung cancer: *“a lot of people would associate it (lung cancer) with smoking… And sort of self-inflicted” (P10*, *decliner*, *female)*

Despite all participants having a smoking history they acknowledged that the idea of lung cancer being self-inflicted also carried over to those without a smoking history. One participant said: *“And then people do get lung cancer that have never smoked*. *And I feel like every time that happens*, *they say ‘oh I never smoked*, *I never smoked’ because they feel that condemnation of society*: *Well*, *you brought this on yourself*.*” (P18*, *screener*, *female)*. Further, this participant’s perception was that lung cancer was different to other cancers because of this self-blame: *“Nobody blames you for getting breast cancer*. *My mother had stomach cancer that she has thankfully been in remission for a long time*, *a decade or so*. *Nobody blames you for that*, *right*? *… but I do think there’s a little bit of public shaming that goes on with lung cancer” (P18*, *screener*, *female)*

#### Access to a General Practitioner (GP)

Nearly all participants had registered for LCS in response to a public advertisement for the study. However, when asked, all felt that health professionals play an important role in guiding people at high-risk to screening: *“I think if a GP can say to people*, *well*, *you know*, *with their history*, *why don’t you think about participating in something like that*? *That’s fine*. *I think probably that’s where it should come from” (P33*, *screener*, *female)*. However, across both participant groups, many spoke at length about the challenges of finding a GP in regional areas, the value of having an established relationship with their GP and their own reluctance to engage in talk about prevention because of GP time constraints: *“You rely on your GP to sort of*, *you know*, *go in there and say five minutes*. *You can only tell them one thing*. *If you got a second thing to talk to them about*, *then make another appointment*. *I mean*, *it’s just ridiculous” (P12*, *decliner*, *female)*

## Discussion

**Fig 1:**
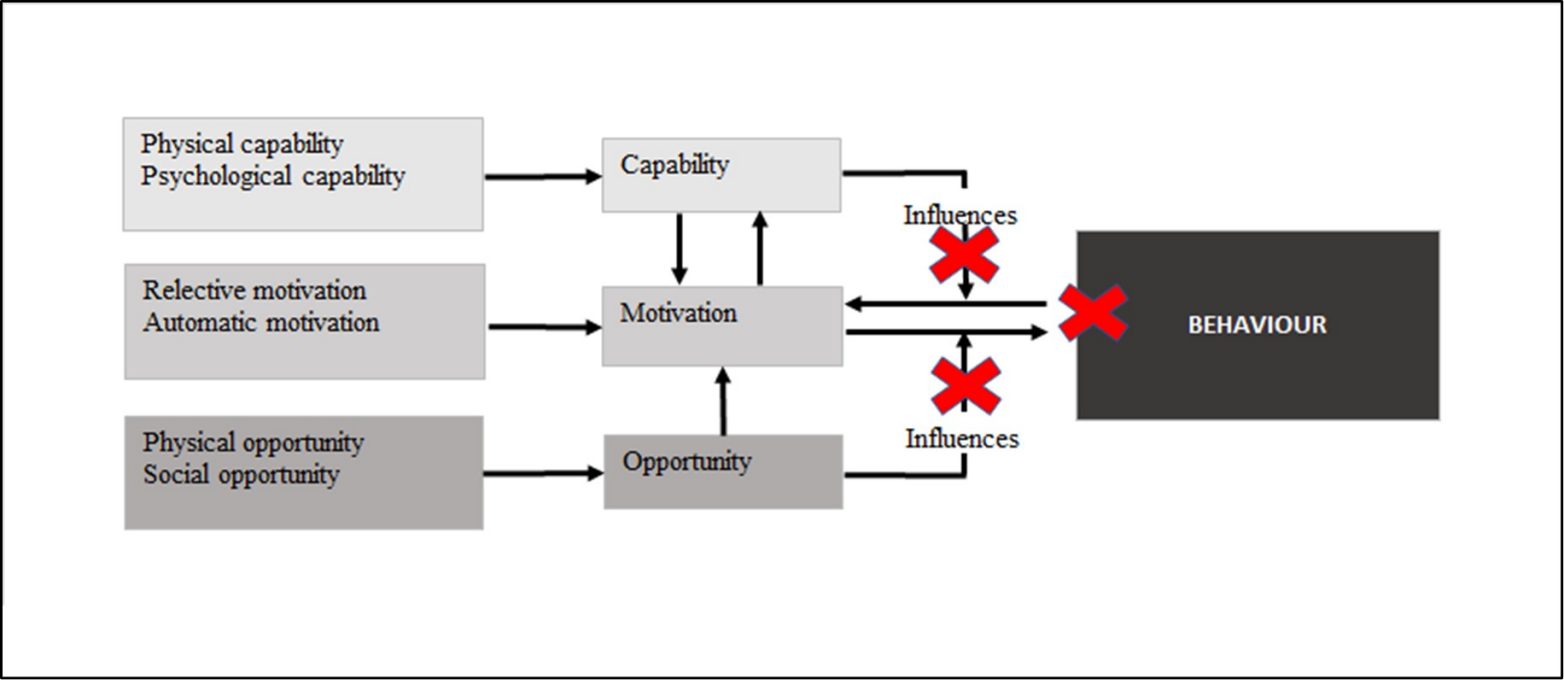
COM-B model: Gates of capability and opportunity adapted from *West et al 2020* [32]. Note the crosses represent a barrier to the opening of the gates of capability and opportunity, reducing the chance motivation will lead to behaviour.

This study aimed to identify key factors that drive LCS participation in the Australian setting to inform future implementation. We interviewed people who had been assessed as high-risk and eligible for a screening trial, some of whom attended screening and some of whom did not. Motivation to participate in screening was high in both groups. The key motivating factors were similar across groups related to the lived experience of lung cancer and a belief in the value of screening, however decliners reported low self-efficacy compared to screeners. Using the COM-B model, our findings suggest that motivation alone may not result in changing screening behaviour unless capability and opportunity are also present [33] (see Fig 1). Decliners, unlike their screening counterparts, reported challenges in both capability and opportunity. ‘Ability to attend’ and ‘knowledge and understanding’ were key themes impacting on decliners capability to participate in screening. Challenges in opportunity for decliners were associated with ‘location as a barrier’ and ‘lack of family support’. These challenges help to explain decliners behaviour highlighting the multiple factors, experienced at the individual level that impact on screening participation. Often these factors are external to the person; identifying these factors may allow targeted strategies that connect the individual with a LCS program such as clear and timely communications, multiple screening sites in convenient locations and accessible helplines, to enhance screening participation.

This is the first study to consider the Australian context, which is important because of the unique aspects of Australia’s diverse aging population concentrated in a few major cities but with a quarter living in outer regional, rural remote areas spread over a large geographical area. Overwhelmingly the challenges associated with the screening location, in particular the distances travelled, out-of-pocket expenses, finding accommodation and unfamiliarity with the site were reported as key barriers by decliners. This may be particularly relevant in the Australian context due to its geography and that the ILST trial centres were located in capital cities, however this has also been reported from the US and UK [10, 15]. Mobile lung screening programs conducted in the US [37] and the UK [38] have demonstrated significant success in recruiting ‘hard-to reach’ communities and overcoming challenges of geography. This delivery model is used by the Australian national screening program for breast cancer [39] and the potential for mobile lung screening vans in Australia has been recognised as a priority [2]. Policy options for the delivery of centralised, decentralised or hybrid models have been identified for UK screening programs but are yet to be widely studied or understood in other jurisdictions [40]. A hybrid approach of community outreach and regional medical centres, with centralized CT scan interpretation, has been recommended as a sustainable mobile program in the UK [37, 40]. Providing options to attend any screening centre, rather than a designated one, and advocating for LCS to meet the eligibility criteria for existing patient financial support schemes are potential strategies to encourage participation.

When prompted to reflect on who encouraged or discouraged attendance, most participants were quick to highlight they were not influenced by others. However, decliners frequently reported challenges with absence of support from a family member for decision-making and enabling transport. Family support is not largely reported as a predictor of participation, but high self-efficacy has been found to be associated with family support [18]. Self-efficacy may have direct implications as a predictor and a mediator of LCS behaviour [18]. In our study, decliners often reported feeling overwhelmed by the prospect of travel to an unfamiliar setting and low personal control in managing their health included disappointment with previous attempts to give up smoking. While low self-efficacy may be an anticipated finding in the high-risk population who are ‘hard to reach’ it remains challenging to build individual’s belief in their capacity to undertake screening in a population setting. Recently, innovation to increase screening attendance following a telephone-based delivery of decision support via an online tool has been reported [41]. Additionally, a reminder letter providing a pre-scheduled appointment increased uptake of LCS among non-responders in a UK trial, although there were no statistically significant differences across control and intervention groups [42]. Acknowledging the role of support and investing in pre-screening support, pre-invitation and reminder letters and online or telephone helplines might help to connect the individual to the program increasing social opportunity to attend screening.

Clinicians have an important role in helping patients make decisions around screening [43, 44]. Decision support is a key opportunity for clinicians to tailor conversations and provide meaningful discussions [18] to mitigate challenges related to self-efficacy. Clinician-targeted interventions including point-of-care information will support the shared decision-making process [18]. However, time constraints and limited access to GPs and health services outside of major cities in Australia, reported by many participants in this study, is well documented [45]. Providing funding for health professionals, in particular GPs and specialist nurses, to spend the time and resources to assist patients in decision-making may help to overcome this barrier to best practice [46]. Recent findings from the SUMMIT study have demonstrated that telephone-based risk assessment administered by technically trained staff was an efficient way to optimise selection for LCS invitations in the UK setting [47]. Furthermore, an increase in the adoption of telehealth services during the COVID-19 pandemic in Australia highlights the potential role of telehealth in improving access to health professionals in regional and remote areas [48].

The highly stigmatised nature of smoking was mentioned throughout all interviews, in relation to social opportunity, though its impact on screening behaviour was not clear. Study participants expressed their concern for people who smoke and may feel the shame of developing lung cancer, so often labelled a “self-inflicted” disease. Raising awareness in both the community and health professionals about the benefits of LCS and challenging outdated ideas of blaming the person diagnosed with lung cancer may provide a first step in reframing screening as a positive public health practice.

A key strength of this study is the use of a theoretical basis to underpin the interview schedule and data analysis. We chose the COM-B model to identify key target points at a given time rather than the intervention functions of the complementary Behaviour Change Wheel [33]. The COM-B model enabled focus on behavioural motivation, capability and opportunity that could ultimately be linked to screening decisions and potential points for intervention. While beliefs about the value of screening appeared strong in screeners and decliners, barriers associated with self-efficacy, an element of motivation, as well as a range of factors in capability and opportunity were differentially voiced, impeding screening behaviour at that moment in time. This may explain the success of interventions such as mobile screening programs that minimise the need for plans, sidestepping these barriers and opening the gate of opportunity (Fig 1) by providing a LDCT scan at the time of identifying high risk.

Another strength of this study was the opportunity to compare the views of screeners and decliners. Key similarity across groups was the relatively high level of motivation to attend screening. Themes including fatalism, awareness of own risk and access to a general practitioner were all identified, but differences between screeners and decliners related perceptions were not evident. However, the groups differed in self-efficacy and personal control (decliners demonstrated lower levels). The association of self-efficacy with other motivation factors in decliners needs consideration in targeting interventions. In a previous study, the link between screening behaviour and self-efficacy appeared to be fully mediated by a number of factors including fatalism and knowledge of lung cancer risk [18].

Decliners reported challenges of distance associated with the screening location and cost (where screeners did not) and a lack of family support in decision making and transport and accommodation for screening. A small number of decliners also showed a poorer understanding than screeners of the purpose of screening and the test used. Many of these differences are modifiable factors if targeted appropriately. We were able to track participant’s consent rates and unsurprisingly it was more difficult to recruit decliners than screeners in this study. This demonstrates the challenges of being able to understand what drives this behaviour in different groups.

A limitation of this study was that participants self-nominated and registered for the ILST indicating a level of motivation. In addition, although the socio-economic status of participants was just below the national average, decliners recruited from Sydney reported a higher socio-economic status than screeners, with 2 of the 5 decliners in this group from the highest quintile [SEIFA score]. These factors may partially explain the lack of observed differences between screeners and decliners in perceived benefits of lung cancer screening, and beliefs around fatalism that have been negatively associated with screening participation in a number of studies [18, 21, 49]. Future studies exploring screening participation should focus on the impact of socio-economic characteristics of individuals in the Australian setting.

Further limitations include that participants’ claims of having sufficient knowledge about screening to support decision making were not confirmed through completion of a validated instrument and so cannot be considered a key factor in driving participation at this stage. In addition, this study did not capture those at high-risk of lung cancer who never approached the ILST study. This is an important group to understand; we postulate they may have low levels of capability and opportunity and motivation. Should this be the case, our findings regarding capability and opportunity could be equally relevant to this group. This study does not take into account what motivates people to continue to attend screening after the first LDCT scan. Future research should investigate how decisions are made in this context.

Increasing the uptake of screening behaviour may demand multi-level approaches and our findings allow for the development of targeted strategies[20]. Future studies that evaluate the impact of interventions on screening behaviour would be a practical next step to guide implementation such as a multi-level strategy to provide pre- and post- screening support, opportunities for decision making and identification of individual challenges.

## Conclusion

We found a high level of motivation to participate in LCS for individuals at high-risk of lung cancer participating in a trial, driven by the lived experience of lung cancer and a belief in the value of screening. Findings from this study indicate that interventions for real-world programs should focus on the practical barriers of capability and opportunity, and on self-efficacy related to motivation rather than trying to increase motivation more broadly. For example, providing online and telephone pre-screening decision making and follow-up support, mobile screening programs to improve accessibility, and funding for clinicians to support individuals in decision-making would contribute to increasing participation in LCS, particularly if given at an appropriate point in time. Targeted interventions that connect the individual with the LCS program will be crucial for successful implementation and further studies in the Australian setting are urgently needed to ensure policy decisions are feasible and appropriate for the high-risk population.

## Supporting information

S1 File Interview guide: Motivation to participate in lung cancer screening. (DOCX)

S1 Table Coding framework with definitions. (PDF)
