# Supplementary material for: Motivation is not enough: A qualitative study of lung cancer screening uptake in Australia to inform future implementation
Source: PLoS One. 2022 Sep 30;17(9):e0275361. doi: 10.1371/journal.pone.0275361 (PMC9524683; doi:10.1371/journal.pone.0275361)
Supplement: S1 File — (DOCX) [file pone.0275361.s001.docx]

**S1 File: Interview Guide *Motivation to participate in lung cancer screening***

For participants who attended screening (screeners) and who declined to attend screening (decliners)

| **FLOW OF DISCUSSION AND KEY QUESTIONS** | **PROMPTS** |
| --- | --- |
| **Introduction** |  |
| *Recently you participated in the Lung Cancer Screening Trial*   1. Can you tell me a bit about your experience of any cancer screening programs that you been involved in? | - 1. What was it like? |
| **Initial reasons** |  |
| 1. Can you talk me through your reasons for taking part (decliners: or signing up for) in lung cancer screening initially? | a. Why were you interested?  b. Has anyone in the family had lung cancer? |
| **Attitudes to lung cancer screening** |  |
| 1. How well do you think screening works in finding lung cancer? | - 1. Do you think it works?   b. Can you tell me a bit more about why you think this? |
| **Motivations and barriers** |  |
| Screeners:   1. What were the factors that encouraged you to have a lung cancer screening test? 2. Were there any factors that made it difficult for you to go for a lung cancer screening test   Decliners:   1. What were the factors that discouraged you to have a lung cancer screening test? 2. Was there anything that encouraged you? | a. What about Convenience; Travel required; Time taken; Cost;  b. Anything about the test itself? (potential complications)  c. Any other factors? |
| **Impact of health professionals** |  |
| 1. Can you tell me about any conversations you might have had with your GP about lung cancer screening? 2. How important is it to you that a health professional recommends cancer screening? | a. What about other health professionals, like a specialist? |
| **Impact of others** |  |
| 1. What did your friends and family think about you going for lung cancer screening?    - - How do you feel about that? 2. Did anyone discourage you?    - - How did you feel about that? | 1. Did you discuss it with them, what did they think about you going/not going, how did they influence your decision 2. Anyone else? (for example Health professional, family, friends) 3. How do you think other people’s views have affected you (and your decision to be screened)? |
| **Impact of stigma** |  |
| 1. How do you think other people [in the community, family and doctors] feel about lung cancer?    - - about screening for lung cancer?      - about smoking? 2. How do you think lung cancer is different to other cancers like breast or bowel cancer or melanoma? |  |
| **Harms and benefits of lung cancer screening** |  |
| 1. Thinking back to before the screening, what did you think were the potential benefits and harms for you of having lung cancer screening? |  |
| **Perception about individual risk and lung cancer** |  |
| 1. How do you feel about lung cancer generally?   - How do you feel about your risk of lung cancer?  - How much do you worry about getting lung cancer? |  |
| **Personal control** |  |
| 1. How confident do you feel in your ability to reduce your risk of lung cancer? | a. How much [personal] control do you think you have in:  - stopping smoking? (if applicable)  - carrying our screening recommendations |
| **Concluding question:**  **Interviewer:** *I have a big picture question to finish* |  |
| 1. Can you tell me about anything that might help to increase the chance that others might attend lung cancer screening? |  |

*That’s everything I wanted to talk to you about today. Is there anything else you’d like to say or discuss? Thank you so much for taking the time to talk with me.*
