# Supplementary material for: Motivation is not enough: A qualitative study of lung cancer screening uptake in Australia to inform future implementation
Source: PLoS One. 2022 Sep 30;17(9):e0275361. doi: 10.1371/journal.pone.0275361 (PMC9524683; doi:10.1371/journal.pone.0275361)
Supplement: S1 Table — (PDF) [file pone.0275361.s002.pdf]

S1 Table: Coding framework with definitions: Drivers of lung cancer screening participation in Australia using the COM-B (capability, opportunity, motivation-behaviour) model

COM-B construct	Construct definition[1]	Framework definitions[2]	Themes
**Capability:**	Individual's capacity to engage in the activity concerned	Physical capability: physique and musculoskeletal functioning capacity	Ability to attend[a]
		Psychological capability: capacity to engage in the necessary thought processes	Knowledge and understanding[a]
**Motivation**	Brain processes that energize and direct behaviour	Automatic processes: involving emotions and impulses that arise from associative learning and/or innate dispositions	Impact of lived experience<br>Fatalism
		Reflective processes: involving evaluations and plans	Awareness of own risk<br>Screening as beneficial<br>Self-efficacy[a]
**Opportunity**	Factors that lie outside the individual that make the behaviour possible or prompt it	Physical opportunity afforded by the environment	Location as a barrier[a]
		Social opportunity afforded by the cultural milieu that dictates the way that we think about things	Support from family[a]<br>Stigma is ever present<br>Access to a General Practitioner

1. Michie S, van Stralen MM, West R. The behaviour change wheel: a new method for characterising and designing behaviour change interventions. Implement Sci. 2011;6:42.
2. West R, Michie S. A brief introduction to the COM-B model of behaviour and the PRIME theory of motivation. Qeios. 2020.

a. decliners and screeners differ
